# Signet-Ring Cell Carcinoma as an Independent Prognostic Factor for Patients With Urinary Bladder Cancer: A Population-Based Study

**DOI:** 10.3389/fonc.2020.00653

**Published:** 2020-05-15

**Authors:** Di Jin, Shi Qiu, Kun Jin, Xianghong Zhou, Qi Cao, Lu Yang, Qiang Wei

**Affiliations:** Department of Urology, Institute of Urology, National Clinical Research Center for Geriatrics and Center of Biomedical Big Data, West China Hospital of Sichuan University, Chengdu, China

**Keywords:** signet ring cell, carcinoma, urinary bladder, SEER, prognosis

## Abstract

**Background:** Primary signet-ring cell carcinoma (SRCC) is a rare variation of adenocarcinoma. Although SRCC of the urinary bladder is highly malignant, it is often neglected due to its rarity.

**Materials and Methods:** We used the national Surveillance, Epidemiology, and End Results (SEER) database (2004–2016) to compare SRCC with urothelial carcinoma (UC) and investigated the prognostic values of the clinicopathological characteristics and survival outcomes in SRCC of the urinary bladder. Multivariable Cox proportional hazard model, subgroup analyses, and propensity score matching (PSM) were used.

**Results:** In all, 318 patients with SRCC and 57,444 patients with UC were enrolled. Compared with those with UC, patients with SRCC were younger at diagnosis (*P* < 0.001) and had higher rates of muscle invasive disease (*P* < 0.001), lymph node metastasis (*P* < 0.001), and distal metastasis (*P* < 0.001), as well as higher-grade tumors (*P* = 0.004). A Cox proportional hazard regression analysis showed that the SRCC group was associated with significantly higher risks of overall mortality (OM) compared with the UC group [hazard ratios (HR) = 1.44, 95% confidence intervals (95% CI) = 1.26–1.63, *P* < 0.0001]. Patients with SRCC also had a higher risk of cancer-specific mortality (CSM; HR = 1.40, 95% CI = 1.18–1.65, *P* < 0.0001). After PSM, the SRCC group also experienced higher risks of OM (HR = 1.45, 95% CI = 1.24–1.68, *P* < 0.0001) and CSM (HR = 1.47, 95% CI = 1.20–1.79, *P* = 0.0001) compared with the UC group. In the subgroup analyses, no significant interactions were observed in sex, age, N stage, M stage, and lymph nodes removed in terms of both OM and CSM.

**Conclusion:** The prognosis of SRCC is poorer than that of UC, even after adjustment for baseline demographic and clinicopathological characteristic as well as cancer treatment. SRCC is an independent prognostic factor for patients with urinary bladder cancer.

## Introduction

Bladder cancer is one of the most common tumors and is a significant cause of tumor-related death worldwide (1). Its worldwide age-standardized incidence rate (per 100,000 person/years) is 9.0 for men and 2.2 for women (2). Although etiological factors have been identified for bladder cancer, most have not been used successfully to reduce its prevalence (3). The most common pathological type is urothelial carcinoma (UC); thus, treatments for bladder cancer primarily focus on UC. Among the bladder cancer types, primary signet ring cell carcinoma (SRCC) is a rare variation of adenocarcinoma, as it accounts for ~0.24% of all bladder malignancies (4). Compared with UC, SRCC tends to present with high-grade disease according to several studies. As a result, the presence of signet ring cells is often associated with a worse prognosis (5).

Thus far, although SRCC of the urinary bladder is highly malignant, it is often neglected due to its rarity. Therefore, this study comprehensively compared SRCC with UC, which represents the most common pathological subtype of urinary bladder cancer (6). We used the national Surveillance, Epidemiology, and End Results (SEER) database (2004–2016) to investigate the prognostic values of the clinicopathological characteristics and survival outcomes in SRCC of the urinary bladder.

## Materials and Methods

### Data Resource and Study Population

Adult patients (≥18 years of age) who were registered from 2004 to 2016 in the SEER database were selected. The primary cancer site was restricted to the urinary bladder (C67) according to the International Classification of Disease for Oncology, Third Edition. Patients were included only if the histology was SRCC (ICD-O-3 8490/3) or UC. UC included ICD-O-3 8120/3 (transitional cell carcinoma), ICD-O-3 8122/3 (transitional cell carcinoma, spindle cell), and ICD-O-3 8131/3 (transitional cell carcinoma, micropapillary). The diagnosis was confirmed by positive histology and was their first or only cancer diagnosis (first positive indicator of malignancy). Patients without histological diagnosis and survival data were excluded.

### End Points

The main end points were overall mortality (OM) and cancer-specific mortality (CSM) according to data in the SEER database. OM refers to deaths from any cause, while CSM is defined as death from SRCC or UC according to the recorded cause of death. Survival time was the duration from initial diagnosis to death from any cause or to the last follow-up.

### Statistical Analysis

Baseline characteristics were assessed to determine whether there were significant differences in the distribution of the study population. Two-sample *t*-tests, Pearson's chi-square tests, and Fisher's exact tests were performed for continuous variables and categorical variables, as appropriate. Kruskal–Wallis H tests were used for non-Gaussian distributions. Continuous variables were presented as the mean ± SD. For age at diagnosis and survival (in months), medians and interquartile ranges were also reported. Categorical variables were shown as frequencies and their proportions. The OM and CSM of each histological subtype were compared using unadjusted Kaplan–Meier curves and the log-rank test.

The multivariable Cox proportional hazard model was used to calculate hazard ratios (HR) and their 95% confidence intervals (95% CI) stratified by histological types. The following covariates were adjusted: sex, age at diagnosis, treatment modality (surgery and radiation), and TNM stage. Subgroup analyses were performed by multivariate regression analysis. Sex, primary site, TNM stage, and lymph nodes removed were adjusted in the Cox model. Tests to determine interactions were also used in the subgroup analyses. Propensity score matching (PSM) was used to further adjust the model for potential baseline confounding factors. All analyses were performed with the statistical software packages R (http://www.R-project.org, The R Foundation) and EmpowerStats (http://www.empowerstats.com, X&Y Solutions, Inc., Boston, MA).

## Results

### Baseline Characteristics of the Study Population

In all, 57,762 patients, including 57,444 patients with UC and 318 patients with SRCC, were identified in the SEER database from 2004 to 2016. Table 1 includes the baseline characteristics of these patients. SRCC patients were younger at diagnosis compared with UC patients (SRCC 66.77 ± 12.74 vs. UC 72.62 ± 11.53, *P* < 0.001). The majority of patients were males in both the UC (75.39%) and SRCC (72.96%) groups, but no difference was found in the proportion of males or females between the two groups (*P* = 0.315). The SRCC group presented with a more advanced stage than the UC group, as shown by a higher rate of muscle invasive disease (71.38 vs. 45.18%, *P* < 0.001), lymph node metastasis (27.99 vs. 8.57%, *P* < 0.001), and distal metastasis (19.50% vs. 7.12%, *P* < 0.001). Higher-grade disease was more common in the SRCC group (98.57 vs. 88.86%, *P* = 0.004). The SRCC group had a lower rate of surgery than the UC group (83.65 vs. 91.32%, *P* < 0.001), whereas lymph nodes were more likely to be removed in the SRCC group than in the UC group (42.14 vs. 20.80%, *P* < 0.001). Moreover, when the type of radiation therapy was known, external beam radiation was more frequently used in the SRCC group than in the UC group (19.18 vs. 9.76%, *P* < 0.001).

**Table 1:** Baseline demographic and clinicopathologic characteristics of patients with SRCC compared to UC.

	***SRCC (n = 318)***	***UC (n = 57,444)***	***P-value***
Mean age (years, SD)	66.77 ± 12.74	72.62 ± 11.53	<0.001
Median age (years, IQR)	68.00 (57.00–76.75)	74.00 (65.00–81.00)	<0.001
Sex			0.315
Male	232 (72.96%)	43,306 (75.39%)	
Female	86 (27.04%)	14,138 (24.61%)	
Marital status			0.001
Married	177 (55.66%)	32,717 (56.95%)	
Single	55 (17.30%)	6,117 (10.65%)	
Widowed/divorced	67 (21.07%)	14,740 (25.66%)	
Unknown	19 (5.97%)	3,870 (6.74%)	
Race			<0.001
White	256 (80.50%)	50,539 (87.98%)	
Black	42 (13.21%)	3,868 (6.73%)	
Other	19 (5.97%)	2,685 (4.67%)	
Unknown	1 (0.31%)	352 (0.61%)	
Year of diagnosis			0.560
2004	23 (7.23%)	4,019 (7.00%)	
2005	28 (8.81%)	3,956 (6.89%)	
2006	24 (7.55%)	4,027 (7.01%)	
2007	20 (6.29%)	4,151 (7.23%)	
2008	31 (9.75%)	4,288 (7.46%)	
2009	15 (4.72%)	4,376 (7.62%)	
2010	30 (9.43%)	4,622 (8.05%)	
2011	27 (8.49%)	4,627 (8.05%)	
2012	26 (8.18%)	4,690 (8.16%)	
2013	19 (5.97%)	4,632 (8.06%)	
2014	25 (7.86%)	4,758 (8.28%)	
2015	26 (8.18%)	4,678 (8.14%)	
2016	24 (7.55%)	4,620 (8.04%)	
Primary Site			<0.001
Trigone of bladder	20 (6.29%)	3,570 (6.21%)	
Dome of bladder	26 (8.18%)	2,571 (4.48%)	
Lateral wall of bladder	34 (10.69%)	9,551 (16.63%)	
Anterior wall of bladder	6 (1.89%)	1,545 (2.69%)	
Posterior wall of bladder	20 (6.29%)	5,015 (8.73%)	
Bladder neck	10 (3.14%)	2,022 (3.52%)	
Ureteric orifice	2 (0.63%)	1,265 (2.20%)	
Urachus	13 (4.09%)	11 (0.02%)	
Overlapping lesion of bladder	49 (15.41%)	7,548 (13.14%)	
Bladder, NOS	138 (43.40%)	24,346 (42.38%)	
T stage			<0.001
Ta	0 (0.00%)	3,463 (6.03%)	
Tis	3 (0.94%)	7,450 (12.97%)	
T1	41 (12.89%)	14,147 (24.63%)	
T2	75 (23.58%)	16,609 (28.91%)	
T3	53 (16.67%)	4,986 (8.68%)	
T4	99 (31.13%)	4,360 (7.59%)	
Unknown	47 (14.78%)	6,429 (11.19%)	
N stage			<0.001
N0	182 (57.23%)	45,120 (78.55%)	
N1	38 (11.95%)	2,472 (4.30%)	
N2	50 (15.72%)	2,335 (4.06%)	
N3	1 (0.31%)	115 (0.20%)	
Unknown	47 (14.78%)	7,402 (12.89%)	
M stage			<0.001
M0	220 (69.18%)	47,132 (82.05%)	
M1	62 (19.50%)	4,089 (7.12%)	
Unknown	36 (11.32%)	6,223 (10.83%)	
Grade			0.004
Low	1 (1.43%)	3,025 (11.14%)	
High	69 (98.57%)	24,138 (88.86%)	
Surgery			<0.001
No surgery	52 (16.35%)	4,984 (8.68%)	
TURBT	95 (29.87%)	32,528 (56.63%)	
Partial cystectomy	26 (8.18%)	1,101 (1.92%)	
Radical cystectomy	54 (16.98%)	5,930 (10.32%)	
Pelvic exenteration	6 (1.89%)	148 (0.26%)	
Other	85 (26.73%)	12,753 (22.20%)	
Lymph nodes removed			<0.001
None	184 (57.86%)	45,493 (79.20%)	
More than one	134 (42.14%)	11,951 (20.80%)	
Radiation			<0.001
Beam radiation	61 (19.18%)	5,609 (9.76%)	
Radioactive implants	0 (0.00%)	15 (0.03%)	
Combination of beam and implants	0 (0.00%)	9 (0.02%)	
Radioisotopes	0 (0.00%)	5 (0.01%)	
Radiation unknown	2 (0.63%)	119 (0.21%)	
Performance unknown	255 (80.19%)	51,687 (89.98%)	
Cancer-specific mortality			<0.001
Alive	173 (54.40%)	43,127 (75.08%)	
Dead	145 (45.60%)	14,317 (24.92%)	
Overall mortality			<0.001
Alive	74 (23.27%)	24,571 (42.77%)	
Dead	244 (76.73%)	32,873 (57.23%)	
Survival time (years, SD)	22.28 (27.28)	37.73 (38.54)	<0.001
Survival time (years, IQR)	12.00 (5.00–27.75)	23.00 (7.00–58.00)	<0.001

*SRCC, signet ring cell carcinoma; UC, urothelial carcinoma; IQR, interquartile range; NOS, not otherwise specified; TURBT, transurethral resection of bladder tumor*.

### Survival Analyses

**Figure 1:**
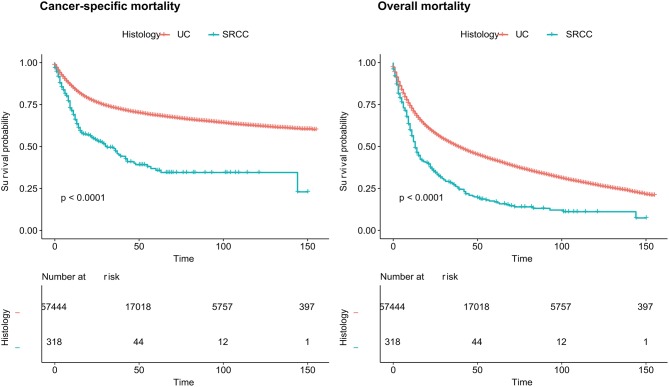
Cancer-specific mortality and overall mortality of patients with SRCC and UC, respectively. SRCC, primary signet ring cell carcinoma; UC, urothelial carcinoma.

According to the survival analyses, the landmark OM of the SRCC population was higher than that of the UC population (*P* < 0.0001) (Figure 1). When the landmark was set to 5 years (60 months), survival probability of the SRCC group also declined more quickly according to the OM analyses but was not obviously different from the SRCC group when the follow-up period was longer than 5 years (Supplementary Figure 1). The SRCC group also had a lower survival probability according to the CSM analyses (*P* < 0.0001) (Figure 1).

The non-adjusted and two adjusted models are presented in Table 2. After adjustments for age, sex, TNM stage, primary tumor site, and treatment method, multivariable Cox proportional hazard regression showed that the SRCC group had a significantly higher risk of OM compared with patients with UC (HR = 1.44, 95% CI = 1.26–1.63, *P* < 0.0001). The SRCC group also had a higher risk of CSM (HR = 1.40, 95% CI = 1.18–1.65, *P* < 0.0001). To minimize selection bias, PSM was performed for baseline factors and treatments (Table 3). Most baseline factors were matched except for primary tumor site, surgery, and radiation. Furthermore, we made an extra adjustment to analyze the mismatched baseline factors. Compared with the UC group, the SRCC group faced higher risks of OM (HR = 1.45, 95% CI = 1.24–1.68, *P* < 0.0001) and CSM (HR = 1.47, 95% CI = 1.20–1.79, *P* = 0.0001) (Table 2).

**Table 2:** Multivariable Cox proportional hazard model.

**Outcomes**	**SRCC HR (95% CI)**	***P*-value**
Cancer-specific mortality		
Non-adjusted	2.39 (2.03, 2.81)	<0.0001
Adjust I	1.29 (1.10, 1.53)	0.0021
Adjust II	1.40 (1.18, 1.65)	<0.0001
PSM non-adjusted	1.50 (1.24, 1.82)	<0.0001
PSM adjusted	1.47 (1.20, 1.79)	0.0001
Overall mortality		
Non-adjusted	1.87 (1.65, 2.12)	<0.0001
Adjust I	1.35 (1.19, 1.54)	<0.0001
Adjust II	1.44 (1.26, 1.63)	<0.0001
PSM non-adjusted	1.44 (1.25, 1.67)	<0.0001
PSM adjusted	1.45 (1.24, 1.68)	<0.0001

*SRCC, signet-ring cell carcinoma; HR, hazard ratio; PSM, propensity score matching*.

*Adjusted I model adjusts for sex, age, T stage, N stage, M stage*.

*Adjusted II model adjusts for sex, age, primary site, T stage, N stage, M stage, surgery, radiation*.

*PSM-non-adjusted model adjusts for none*.

*PSM-adjusted model adjusts for surgery, radiation, primary site*.

**Table 3:** Propensity score matching for baseline factors.

	**SRCC**	**UC**	***P*-value**
Mean age (years, SD)	66.65 ± 12.83	66.56 ± 12.43	0.9099
Sex			0.9886
Male	222 (72.3)	885 (72.1)	
Female	85 (27.7)	343 (27.9)	
Marital status			0.4588
Married (including common law)	171 (55.7)	717 (58.4)	
Single	54 (17.6)	180 (14.7)	
Widowed/divorced	65 (21.2)	277 (22.6)	
Unknown	17 (5.5)	54 (4.4)	
Race			0.0713
White	245 (79.8)	1,040 (84.7)	
Black	42 (13.7)	116 (9.4)	
Other	19 (6.2)	60 (4.9)	
Unknown	1 (0.3)	12 (1)	
Primary site			<0.0001
Trigone of bladder	20 (6.5)	71 (5.8)	
Dome of bladder	26 (8.5)	52 (4.2)	
Lateral wall of bladder	33 (10.7)	188 (15.3)	
Anterior wall of bladder	6 (2)	34 (2.8)	
Posterior wall of bladder	19 (6.2)	76 (6.2)	
Bladder neck	10 (3.3)	46 (3.7)	
Ureteric orifice	1 (0.3)	26 (2.1)	
Urachus	13 (4.2)	0 (0)	
Overlapping lesion of bladder	49 (16)	204 (16.6)	
Bladder, NOS	130 (42.3)	531 (43.2)	
T stage			0.2939
Ta	0 (0)	8 (0.7)	
Tis	3 (1)	18 (1.5)	
T1	37 (12.1)	146 (11.9)	
T2	74 (24.1)	304 (24.8)	
T3	52 (16.9)	214 (17.4)	
T4	95 (30.9)	310 (25.2)	
Unknown	46 (15)	228 (18.6)	
N stage			0.2626
N0	176 (57.3)	764 (62.2)	
N1	37 (12.1)	117 (9.5)	
N2	49 (16)	152 (12.4)	
N3	1 (0.3)	6 (0.5)	
Unknown	44 (14.3)	189 (15.4)	
M stage			0.3518
M0	214 (69.7)	891 (72.6)	
M1	59 (19.2)	194 (15.8)	
Unknown	34 (11.1)	143 (11.6)	
Grade			0.2945
Low	1 (1.5)	31 (5.4)	
High	64 (98.5)	545 (94.6)	
Surgery			<0.0001
no surgery	48 (15.6)	131 (10.7)	
TURBT	91 (29.6)	478 (38.9)	
Partial cystectomy	26 (8.5)	29 (2.4)	
Radical cystectomy	53 (17.3)	249 (20.3)	
Pelvic exenteration	5 (1.6)	8 (0.7)	
Other	84 (27.4)	333 (27.1)	
Lymph nodes removed			0.3288
None	176 (57.3)	744 (60.6)	
More than one	131 (42.7)	484 (39.4)	
Radiation			0.0036
Beam radiation	59 (19.2)	148 (12.1)	
Radiation unknown	2 (0.7)	5 (0.4)	
Performance unknown	246 (80.1)	1075 (87.5)	
Cancer-specific mortality			0.0003
Alive	164 (53.4)	796 (64.8)	
Dead	143 (46.6)	432 (35.2)	
Overall mortality			<0.0001
Alive	71 (23.1)	461 (37.5)	
Dead	236 (76.9)	767 (62.5)	

*SRCC, signet ring cell carcinoma; UC, urothelial carcinoma; NOS, not otherwise specified; TURBT, transurethral resection of bladder tumor*.

### Subgroup Analyses

**Figure 2:**
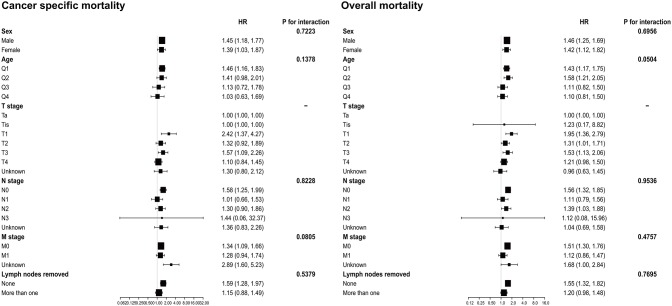
Subgroup analysis for interaction between SRCC and potential covariates in both overall mortality and cancer-specific mortality. SRCC, primary signet ring cell carcinoma.

The results of the subgroup analyses are shown in Figure 2. After adjusting for potential covariates, the tests for interaction were not statistically significant for sex, age, N stage, M stage, and lymph nodes removed in both OM and CSM. The results also indicated that patients with SRCC had a worse prognosis out of all the groups.

## Discussion

In this study, we aimed to investigate the prognostic values of the clinicopathological characteristics and survival outcomes in SRCC of the urinary bladder. Given that UC accounts for ~95% of bladder cancers, SRCC and UC were compared in records from the SEER database under specified inclusion criteria (6). SRCC and UC had differing effects on patients' OM, especially for the 5-year survival status (*P* < 0.0001). Moreover, patients with SRCC had a higher risk of OM (HR = 1.44, 95% CI = 1.26–1.63, *P* < 0.0001) and CSM (HR = 1.40, 95% CI = 1.18–1.65, *P* < 0.0001). The results indicated that SRCC could be an independent prognostic factor for patients with urinary bladder cancer. Furthermore, no interactions were found between sex, age, N stage, M stage, and lymph nodes removed and these two histological subtypes that would influence patient survival.

Given that SRCC was a rare variant, most recent studies were still case reports (7–9). The results of this study support previous studies based on case series and the SEER database before 2004 (10–12). Previous studies suggested that SRCC patients have worse cancer-specific survival than those with UC despite aggressive surgical therapy (10). Compared with the study by Wang et al., the distribution of patient characteristics in this study was similar, but this study included more males with SRCC (75.39%, 232/318 vs. 66%, 68/103). In terms of treatment, our study population had a higher rate of radiation therapy compared with the study by Wang et al. (19.18 vs. 15.5%). In addition, lymph nodes were more frequently removed in the SRCC group than in the UC group (42.14 vs. 20.80%, *P* < 0.001). For TNM stage, higher rates of muscle invasive disease, lymph node metastasis, and distal metastasis of SRCC were confirmed, which could cause a poor prognosis of the SRCC group. Moreover, subgroup analyses indicated that SRCC primarily led to poor prognosis of patients in all subgroups regardless of the age, N stage, M stage, or lymph nodes removed. Interestingly, for the lymph-node-removed subgroup, no difference was observed in either CSM or OM between the SRCC and UC groups. This indicated that removal of lymph nodes might not influence the prognosis of patients with SRCC. To further explore the value of lymph node removal in SRCC, a future study should collect more details regarding the numbers of lymph nodes removed and the number of positive nodes to conduct a stratified analysis.

The majority of SRCC cases in this study were muscle invasive bladder cancer (MIBC), while most bladder cancers do not involve the muscle wall of the bladder (13). SRCC is highly malignant, dedifferentiated, and aggressive, but its mechanism is not yet clear. Fukui Y assumed that an abnormally activated ErbB2/ErbB3 complex can enhance cell growth and lead to the loss of adherence and tight junctions (14). Thomas et al. analyzed immunohistochemical markers in the signet ring component of bladder adenocarcinomas and suggested that component was associated with invasion of the surrounding tissue and lymphatic metastasis (15). Loss of E-cadherin and MUC4 is important for SRCC malignancy (16, 17). The proportion of the SRCC component is associated with advanced-stage disease and worse outcomes. Its invasion pattern could spread laterally and widely without protruding through the surface (18). Cystoscopy might only reveal edematous, bullous, or erythematous mucosa (19). Therefore, rapid infiltration of the submucosa and a lack of obvious mucosal lesions in the early stage could lead to inappropriate staging under cystoscopy without full-thickness biopsy and allow SRCC more time to progress.

The most common surgery in the SRCC group in this study was transurethral resection of bladder tumor (95/318), and the second was radical cystectomy (54/318). However, according to the National Comprehensive Cancer Network guidelines, MIBC without metastasis should be evaluated for radical cystectomy (20). Given that this study enrolled 318 patients with SRCC, their treatments were relatively conservative and might be responsible for their worse prognosis. Other possible explanations were put forward to explain why fewer patients than expected may have undergone surgery. Some older patients might have had a poorer health condition and could not undergo surgery. For patients with metastasis, surgery might not be the standard treatment, and consequently, they may be considered for palliative therapy instead of radical cystectomy. Patients with MIBC were usually treated with systemic chemotherapy, cystectomy, or radiotherapy, and those whose cancer is too advanced to be cured may receive radiotherapy and systemic chemotherapy. However, the SEER database does not provide information on chemotherapy, which was an obvious weakness of our study. Although some studies found that SRCC was resistant to systematic chemotherapy based on small sample sizes (18), some recent studies reported that adjuvant chemotherapy may be a potential component of effective treatment (21, 22). Checkpoint-inhibitor drugs and genomic research offer hope to improve the treatment of bladder cancer (23). We suggest that once SRCC components are found by biopsy, an advanced combined treatment should be considered, but multicenter clinical trials are needed to establish a better therapeutic protocol for this rare but aggressive cancer.

Our study also had some strengths. First, we enrolled 318 patients with SRCC of the urinary bladder who presented from 2004 to 2016 and, thus, had sufficient samples to make more exact and multiform analyses. Subgroup analyses and PSM were used to analyze potential confounding factors. Second, we updated the clinicopathological characteristics and survival outcomes of SRCC based on recent data. This study also had some limitations. Selective bias may exist, which is inevitable for clinical observational studies even when PSM is used. Moreover, treatment regimens were classified into two major categories as surgery and radiotherapy, but chemotherapy and new therapies such as checkpoint-inhibitor drugs, which may lead to different outcomes, were not included in the SEER database.

## Conclusion

The prognosis of SRCC is poorer than that of UC, even after adjustment for baseline demographic and clinicopathological characteristics, as well as cancer treatment. SRCC is an independent prognostic factor for patients with urinary bladder cancer.

## Data Availability Statement

Publicly available datasets were analyzed in this study. This data can be found here: https://seer.cancer.gov/data/.

## Author Contributions

All authors contributed to the design of the project, data collection, and analysis, and contributed to the final manuscript. All authors have read and approved the final submitted manuscript.

## Conflict of Interest

The authors declare that the research was conducted in the absence of any commercial or financial relationships that could be construed as a potential conflict of interest.

## Supplementary Material

Supplementary Figure 1 Five-years cut of overall mortality of patients with primary signet-ring cell carcinoma.

## Abbreviations

SRCC: signet ring cell carcinoma
SEER: Surveillance, Epidemiology, and End Results
UC: urothelial carcinoma
PSM: propensity score matching
OM: overall mortality
CSM: cancer-specific mortality
COD: cause of death
IQR: interquartile range
NOS: not otherwise specified
MIBC: muscle invasive bladder cancer.

## References

[1] Antoni S Ferlay J Soerjomataram I Znaor A Jemal A Bray F. Bladder cancer incidence and mortality: a global overview and recent trends. Eur Urol. (2017) 71:96–108. 10.1016/j.eururo.2016.06.010 27370177

[2] Babjuk M Burger M Comperat EM Gontero P Mostafid AH. Palou J. European association of urology guidelines on non-muscle-invasive bladder cancer (TaT1 and carcinoma *in situ*) -. 2019 Update. Eur Urol. (2019). 76:639–57. 10.1016/j.eururo.2019.08.016 31443960

[3] Babjuk M. Trends in bladder cancer incidence and mortality: success or disappointment? Eur Urol. (2017) 71:109–10. 10.1016/j.eururo.2016.06.040 27417034

[4] Torenbeek R Koot RA Blomjous CE De Bruin PC Newling DW Meijer CJ. Primary signet-ring cell carcinoma of the urinary bladder. Histopathology. (1996) 28:33–40. 10.1046/j.1365-2559.1996.262303.x 8838118

[5] Lee WS Chun HK Lee WY Yun SH Cho YB Yun HR. Treatment outcomes in patients with signet ring cell carcinoma of the colorectum. Am J Surg. (2007) 194:294–8. 10.1016/j.amjsurg.2006.12.041 17693269

[6] Al-Husseini MJ Kunbaz A Saad AM Santos JV Salahia S Iqbal M. Trends in the incidence and mortality of transitional cell carcinoma of the bladder for the last four decades in the USA: a SEER-based analysis. BMC Cancer. (2019) 19:46. 10.1186/s12885-019-5267-3 30630456 PMC6327491

[7] Bouhajja L Farah F Garbouj N Rammeh S. Primary signet-ring cell carcinoma of the urinary bladder: A report of two cases. Tunis Med. (2019) 97:167–169. 31535712

[8] Jayarajah U Fernando DMH Herath KB de Silva MVC. Primary signet-ring cell adenocarcinoma of the urinary bladder treated with partial cystectomy: a case report and review of the literature. Case Rep Urol. (2017) 2017:6829692. 10.1155/2017/6829692 29375924 PMC5742440

[9] Lendorf ME Dohn LH B AD Loya AC Pappot H. An updated review on primary signet-ring cell carcinoma of the urinary bladder and report of a case. Scand J Urol. (2018) 52:87–93. 10.1080/21681805.2017.1418020 29291665

[10] Wang J Wang FW Kessinger A. The impact of signet-ring cell carcinoma histology on bladder cancer outcome. World J Urol. (2012) 30:777–83. 10.1007/s00345-011-0718-8 21706144

[11] Akamatsu S Takahashi A Ito M Ogura K. Primary signet-ring cell carcinoma of the urinary bladder. Urology. (2010) 75:615–8. 10.1016/j.urology.2009.06.065 19819534

[12] Grignon DJ Ro JY Ayala AG Johnson DE. Primary signet-ring cell carcinoma of the urinary bladder. Am J Clin Pathol. (1991) 95:13–20. 10.1093/ajcp/95.1.13 1702926

[13] Bladder cancer: diagnosis and management of bladder cancer: (c) NICE (2015) Bladder cancer: diagnosis and management of bladder cancer. BJU Int. (2017) 120:755–65. 10.1111/bju.14045 29168333

[14] Fukui Y. Mechanisms behind signet ring cell carcinoma formation. Biochem Biophys Res Commun. (2014) 450:1231–3. 10.1016/j.bbrc.2014.07.025 25019985

[15] Thomas AA Stephenson AJ Campbell SC Jones JS Hansel DE. Clinicopathologic features and utility of immunohistochemical markers in signet-ring cell adenocarcinoma of the bladder. Hum Pathol. (2009) 40:108–16. 10.1016/j.humpath.2008.06.022 18789486

[16] Yokoyama A Shi BH Kawai T Konishi H Andoh R Tachikawa H. Muc4 is required for activation of ErbB2 in signet ring carcinoma cell lines. Biochem Biophys Res Commun. (2007) 355:200–3. 10.1016/j.bbrc.2007.01.133 17292332

[17] Lim MG Adsay NV Grignon DJ Osunkoya AO. E-cadherin expression in plasmacytoid, signet ring cell and micropapillary variants of urothelial carcinoma: comparison with usual-type high-grade urothelial carcinoma. Mod Pathol. (2011) 24:241–7. 10.1038/modpathol.2010.187 20818341

[18] Wang J Wang FW. Clinical characteristics and outcomes of patients with primary signet-ring cell carcinoma of the urinary bladder. Urol Int. (2011) 86:453–60. 10.1159/000324263 21525723

[19] Kinra P Rashmi SP Alam A Singh H Dash SC. Primary signet cell adenocarcinoma of bladder. Indian J Pathol Microbiol. (2017) 60:584–6. 10.4103/ijpm.ijpm_201_16 29323081

[20] Spiess PE Agarwal N Bangs R Boorjian SA Buyyounouski MK Clark PE. Bladder cancer, version 5.2017, NCCN clinical practice guidelines in oncology. J Natl Compr Canc Netw. (2017) 15:1240–67. 10.6004/jnccn.2017.0156 28982750

[21] Hamakawa T Kojima Y Naiki T Kubota Y Yasui T Tozawa K. Long-term survival of a patient with invasive signet-ring cell carcinoma of the urinary bladder managed by combined s-1 and Cisplatin adjuvant chemotherapy. Case Rep Urol. (2013) 2013:915874. 10.1155/2013/915874 23738191 PMC3664482

[22] Ohtaka M Kawahara T Kumano Y Maeda Y Kondo T Mochizuki T. Invasive urothelial carcinoma, lymphoma-like/plasmacytoid variant, successfully treated by radical cystectomy with adjuvant chemotherapy: a case report. J Med Case Rep. (2016) 10:48. 10.1186/s13256-016-0806-x 26951070 PMC4782328

[23] Grayson M. Bladder cancer. Nature. (2017) 551:S33. 10.1038/551S33a 29117156

