# Supplementary figures and images for: Signet-Ring Cell Carcinoma as an Independent Prognostic Factor for Patients With Urinary Bladder Cancer: A Population-Based Study
Source: Front Oncol. 2020 May 15;10:653. doi: 10.3389/fonc.2020.00653 (PMC7242733; doi:10.3389/fonc.2020.00653)

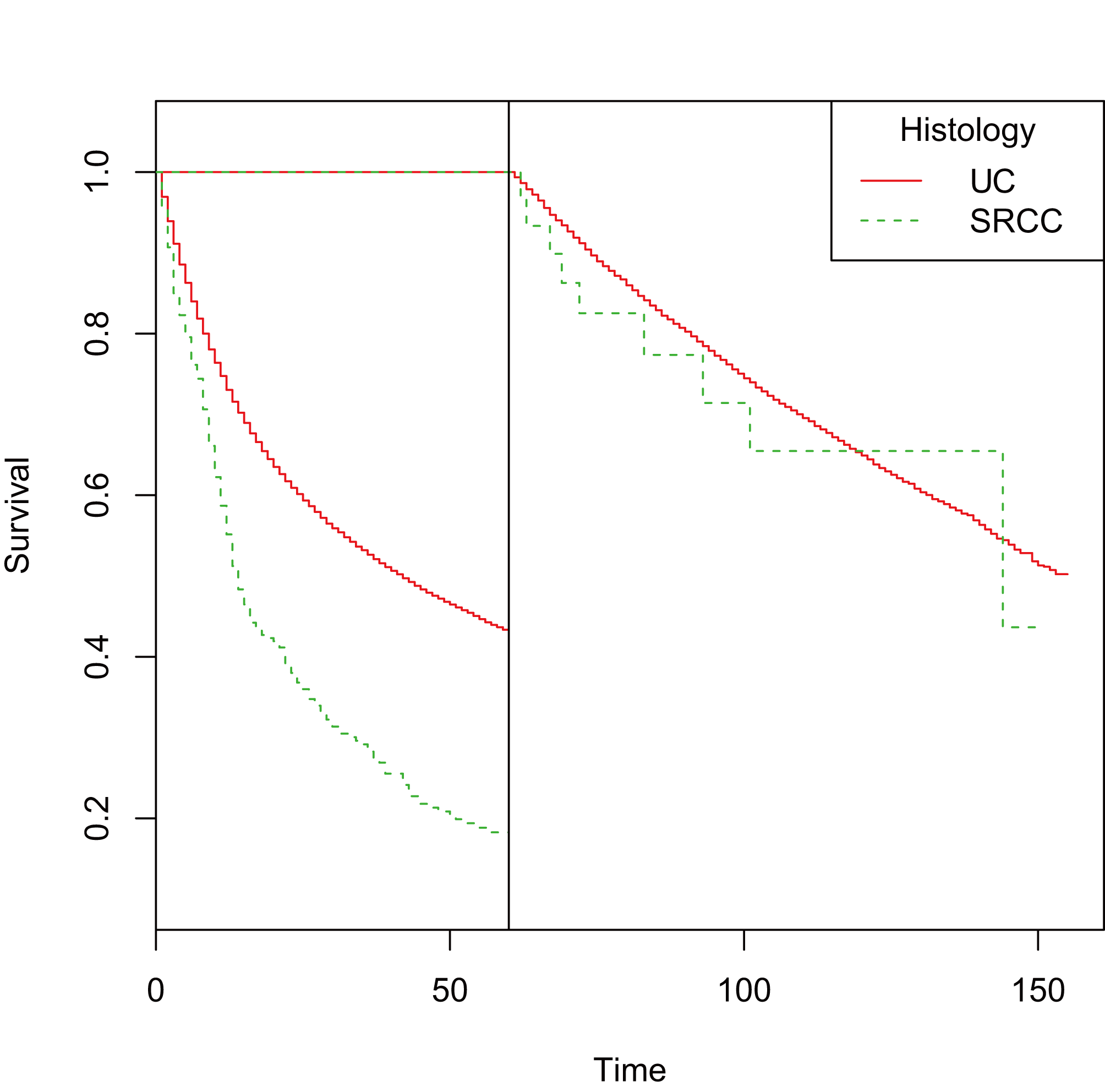

Supplement: Supplementary Figure 1 — Five-years cut of overall mortality of patients with primary signet-ring cell carcinoma. [file Image_1.tif]
